# Eye movement patterns during viewing face images with neutral expressions in patients with early‐stage Alzheimer's disease and amnestic mild cognitive impairment

**DOI:** 10.1002/brb3.3232

**Published:** 2023-08-21

**Authors:** Hatice Eraslan Boz, Koray Koçoğlu, Müge Akkoyun, Işıl Yağmur Tüfekci, Merve Ekin, Gülden Akdal

**Affiliations:** ^1^ Department of Neurosciences, Institute of Health Sciences Dokuz Eylül University Izmir Türkiye; ^2^ Department of Neurology, Unit of Neuropsychology Dokuz Eylül University Izmir Türkiye; ^3^ Department of Neurology Dokuz Eylül University Izmir Türkiye

**Keywords:** Alzheimer's disease, amnestic mild cognitive impairment, eye movements, face processing, visual difficulties

## Abstract

**Background:**

Alzheimer’s disease (AD) neuropathology affects the brain regions responsible for visuospatial skills. Accumulating evidence points to visual difficulties involving face processing in AD and amnestic mild cognitive impairment (aMCI). No study has so far examined eye movement patterns when viewing faces with neutral expressions in patients with AD.

**Aim:**

The objective of this study aimed to examine the eye movements of patients with early‐stage AD, aMCI, and healthy controls (HC) during viewing face images.

**Materials&Methods:**

Thirty‐one AD, 37 aMCI, and 33 HC were included in the study. Eye movements in facial stimuli were recorded with the EyeLink 1000 Plus eye‐tracker.

**Results:**

Our findings showed that AD patients looked less at the eye area of interest than the nose and mouth areas of interest compared to aMCI and HC. Regardless of the group, all participants looked at the eye and nose areas of interest more and longer in the mouth area of interest. In addition, the first fixation duration to the eye area of interest of all participants was shorter than that of the nose and mouth.

**Discussion:**

Consistent with our study, studies in healthy adults revealed eye movement patterns that focused more on the eyes and nose. AD patients are unable to pay attention to the salient parts of faces, tending to focus instead on the non‐informative parts.

**Conclusion:**

Our study is the first to reveal eye movement differences in face processing in AD.

## Graphical abstract

Patients with early‐stage Alzheimer's disease were less interested in eyes and noses on faces with neutral emotional expression than cognitively healthy adults and patients with amnestic mild cognitive impairment. This may be related to the difficulty in visual attention and distinguishing facial identity in these patients. The pattern of viewing facial images of patients with amnestic mild cognitive impairment was not different from healthy adults.

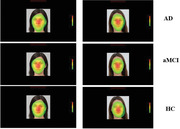

## 1 INTRODUCTION

Alzheimer's disease (AD) has been defined as a clinical and biological entity (McKhann et al., 1984). Three pathophysiological changes, including amyloid beta, fibril tau, and neuronal damage, are accepted as biomarkers of AD. Low levels of Aβ42 in cerebrospinal fluid (CSF) are considered biomarkers of Aβ plaques (Klunk et al., 2004; Mattsson et al., 2009; Villain et al., 2012). High levels of CSF phosphorylated tau (P‐tau) have been identified as biomarkers of fibril tau (Brier et al., 2016; Chhatwal et al., 2016; Mattsson et al., 2009). T‐tau in CSF, hypometabolism on fluorodeoxyglucose‐positron emission tomography (FDG‐PET), and atrophy on magnetic resonance imaging have been identified as biomarkers of neurodegeneration (Besson et al., 2015; Fox et al., 2001; Landau et al., 2011). According to the National Institute on Aging and Alzheimer's Association criteria, dementia in which all biomarkers are positive is called AD with dementia, if all markers are positive, daily functions are preserved but cognitive impairment is defined as AD with mild cognitive impairment (prodromal AD), and if all biomarkers are positive but no cognitive impairment is called preclinical AD. These three states are seen as the AD continuum (Jack et al., 2018). It is stated that CSF markers and amyloid PET are the most specific biomarkers in determining AD (Dubois et al., 2014). Another classification has also been made according to cognitive impairments and it is stated that there is a cognitive continuum in cognitive performance, just as there are biomarkers. This continuum included cognitively unimpaired individuals, mild cognitive impairment, and AD (Lopez et al., 2014; Petersen et al., 2010; Rowe et al., 2010). It is recommended to carry out studies that include participants in the study according to cognitive classification because clinical studies provide increasing cognitive evidence and are less costly compared to biomarker‐based studies (Jack et al., 2018; Mormino et al., 2017).

Researchers point out that the fusiform face area in the fusiform gyrus in the medial temporal lobe responsible for facial identification is affected by AD pathology (Fox et al., 2009; Haxby et al., 2000). Alterations in the anterior medial temporal lobe and fusiform gyrus have also been indicated to occur in patients with amnestic mild cognitive impairment (aMCI) (Whitwell et al., 2007). This evidence contributes to the assumption that face processing may have difficulties in AD and aMCI. Difficulty in face‐processing skills may be an early behavioral hallmark in AD.

Consistent with the neuroimaging findings above, a lot of studies have shown that memory of unfamiliar faces and recognition of facial expressions skills are impaired in AD (Güntekin et al., 2019; Lavallée et al., 2016; Mazzi et al., 2020; Seelye et al., 2009; Spoletini et al., 2008; Werheid & Clare, 2007). In addition, poor face memory and recognition of facial expression performance have been demonstrated in patients with aMCI (Kawagoe et al., 2017; Nguyen et al., 2014; Seelye et al., 2009; Teng et al., 2007). It has been reported that there is a perceptual difference in face‐processing skills in aMCI compared to healthy individuals (Lim et al., 2011).

In the studies mentioned above, pencil‐and‐paper neuropsychological tests, neuroimaging, and electrophysiology methods were used for face processing in AD and aMCI. It is possible to reveal deficits in face processing using eye‐tracking in AD and MCI. Eye movements allow for following the traces of the eyes of the participants with high resolution (Klein & Ettinger, 2019). Fixations in which the eye remains motionless in a particular part of the stimulus and saccades to movements from one point to another constitute eye movement events. Fixations are assumed to focus on the most salient parts of the stimulus and, fixation durations can show the duration of the focus (Henderson & Pierce, 2008). Eye measurements provide objective behavioral measures of visual attention and visual processing (Findlay & Gilchrist, 2003). Providing real‐time results, eye‐tracking technology gives participants the opportunity to clearly track eye movement events during face processing as they view images of faces. Only one study recorded eye movements during face processing in aMCI (Kawagoe et al., 2017). To our knowledge, no study examines eye movement patterns during face viewing in AD. Therefore, studies evaluating face processing in both aMCI and AD will contribute to the understanding of visuospatial difficulties in AD and aMCI, which is considered a precursor to AD. Considering neuroimaging and behavioral evidence, we predict that individuals with AD and aMCI will have different facial scanning patterns during face processing compared to healthy older adults.

Faces form important components of social life. Eye tracking studies conducted with healthy individuals showed increased fixations in the face images, mostly toward the eyes, mouth, and nose (Buswell, 1935; Tatler et al., 2010; Yarbus, 1967). How AD patients scan faces and what parts of faces they pay attention to are essential questions for face‐processing research. Although no eye‐tracking studies are associated with face processing in AD patients, it has been shown that patients lose a lot of time during target search, fail to find the target, and have a more superficial visual scanning pattern compared to controls (Akkoyun et al., 2023; Boz et al., 2023). A more superficial visual scanning pattern has been reported in patients with aMCI than in controls (Kawagoe et al., 2017).

The main aim of this study was to examine eye movement during scanning in neutral face expression in patients with AD and aMCI compared to healthy older individuals. This study aims to analyze eye movement events during face viewing in AD, aMCI patients, and healthy controls (HC). Accordingly, our hypothesis is as follows: since healthy individuals tend to pay attention to facial components such as the eyes and nose, AD and MCI patients will have fewer fixations toward these regions, which will reflect impairments in face processing. To test this hypothesis, we divided the facial stimulus into eye, mouth, and nose areas of interest (IA) and analyzed the number of fixations, fixation durations, and first fixation duration in these areas in AD, aMCI, and older individuals.

## 2 MATERIALS AND METHODS

### 2.1 Participants

The study was performed in Dokuz Eylül University, Neurology Department, Neurosciences Department, and Balance and Eye Movement Recording Laboratory. The participants underwent a neurological examination, and neuropsychological assessment and recording of their eye movements were taken. This study involved 101 participants, comprising 31 individuals with AD, 37 with aMCI, and 33 cognitively HC. The age range for AD, aMCI, and HC was 55–88, 54–79, and 59–90, respectively.

The diagnostic guidelines presented by Albert et al. (2011) and Petersen et al. (2009) for identifying aMCI caused by AD are as follows: First, there must be a decline in cognitive abilities, which is noticeable to either the patient or their family, in comparison to their previous level of functioning. Second, the individual must score at least 1.5 standard deviations below the standardized norms for their age and level of education on a neuropsychological test in at least one cognitive domain. Third, the individual must be capable of performing daily activities independently, without assistance. Lastly, a diagnosis of dementia must be ruled out, and patients with neurological or psychiatric conditions other than aMCI were excluded from the study sample.

To diagnose AD dementia, patients must first fulfill the primary clinical criteria for dementia, which include a decline in functionality compared to their prior levels, difficulty maintaining daily activities, social and occupational functioning, impairment in at least two of the following domains: memory, executive functions, visuospatial functions, or behavior, and the absence of delirium or major depression. The probable criteria for AD dementia are as follows: The cognitive symptoms of the disease should gradually begin, there must be evidence of worsening cognitive impairments, symptoms initially appear either in the form of amnestic (memory impairments) or non‐amnestic (impairments except memory), and there should be no evidence of cerebrovascular disease, other types of dementia, or drug‐related causes that could explain these symptoms (McKhann et al., 2011).

For this study, the researchers chose a control group consisting of individuals who were cognitively healthy. The controls were selected based on two criteria: First, they were no record of neurological or psychiatric disorders in their medical history, and second, they obtained standard scores on neuropsychological tests adjusted for age and education levels.

All study participants either had unaided normal vision or wore corrective lenses like glasses or contact lenses to correct any visual impairments they had. Individuals with hearing impairments that might impede effective communication with investigators were excluded. After providing participants with a thorough explanation of the study, the researchers obtained informed consent from patients or their relatives and from the control group. The study was administered with the approval of the ethics committee of Dokuz Eylül University, in compliance with the guidelines for the declaration of Helsinki (protocol number: 2019/18‐32).

### 2.2 Neuropsychological administration

The Mini–mental state examination is used to detect general cognitive status (Folstein et al., 1975). The cut‐off point for the Turkish population was determined as 23/24 (Keskinoglu et al., 2009).

Verbal and visual memory functions were evaluated with the Öktem Verbal Memory Processes Test and Rey Complex Figures Test (Meyers & Meyers, 1995; Öktem, 1992; Ossterrieth, 1944; Rey, 1941; Varan et al., 2007). Executive functions consisting of the sub‐functions of planning, sequencing, verbal fluency, cognitive flexibility, and inhibition were evaluated with the Wisconsin Card Sorting Test, Stroop Test, phonemic fluency, semantic fluency, and Clock Drawing Tests (Berg., 1948; Heaton, 1981; Karakaş et al., 2013; Martin et al., 1994; Rouleu et al., 1992; Stroop, 1935; Tumaç, 1997; Wechsler, 1987).

Attention was assessed by Digit Span and Trail Making tests (Cangöz et al., 2007; Reitan, 1958; Wechsler, 1987).

Visual‐spatial functions consisting of perception, face recognition, and orientation were evaluated with the Benton Face Recognition Test Benton Line Orientation Judgment (Benton & Van Allen, 1968; Benton et al., 1978; Karakaş, 2006; Keskinkılıç, 2008).

The Boston Naming Test was used to evaluate the language functions of confrontation naming ability (Kaplan et al., 2001; Kurt et al., 2016; Morris et al., 1989).

### 2.3 Apparatus

Eye movements during viewing images were recorded binocularly with the SR Research EyeLink 1000 Plus infrared eye tracking system. The accuracy was up to 0.15°. EyeLink 1000 Plus allows online tracking of eye movement events such as saccade and fixation with a sample rate of 2000 Hz via Host PC and Display PC connected by Ethernet link. Host PC provides real‐time eye data, calibration, and validation monitoring by the experimenter. Display PC provides a presentation of the calibration and validation and trial stimuli.

The size of the display PC (Philips 226E9Q) was 21.5 in. and the aspect ratio was 16:9. The width of the PC was 476 × 268 mm, and the resolution of the screen was 1920 × 1080 pixels. The distance between the participant and the center of the screen was set to 52 cm.

The SR Research Head Support which can be adjusted in height and depth was used for eye movement recording to prevent head and neck movements of the participants and to provide a better head position. Calibration and validation were carried out before eye movement recordings and after the break.

### 2.4 Face paradigm

**FIGURE 1:**
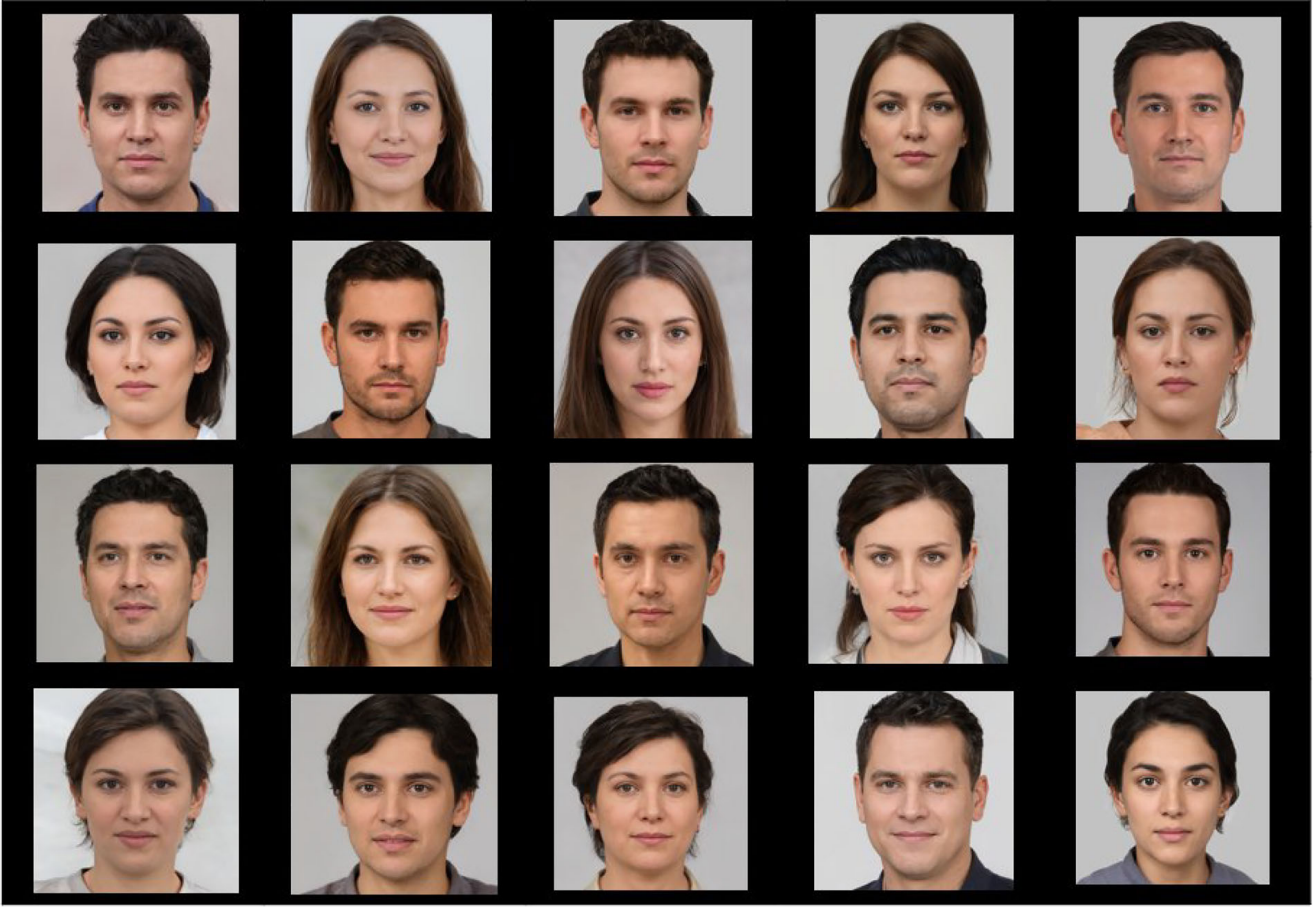
Representation of artificial neutral face stimuli used in the study. The stimuli consisting of images of 10 men and 10 women were randomly displayed to the participants.

The facial stimuli in this study were obtained from the free‐to‐use website “generated.photos” [https://generated.photos/] for research purposes. These stimuli are neutral face images created by artificial intelligence without emotional expression (Figure 1). The size of the stimuli was 10° and 604 pixels. Twenty facial image stimuli, consisting of 10 male and 10 female images, were randomly shown to the participants. Participants were instructed to view the face pictures presented.

**FIGURE 2:**
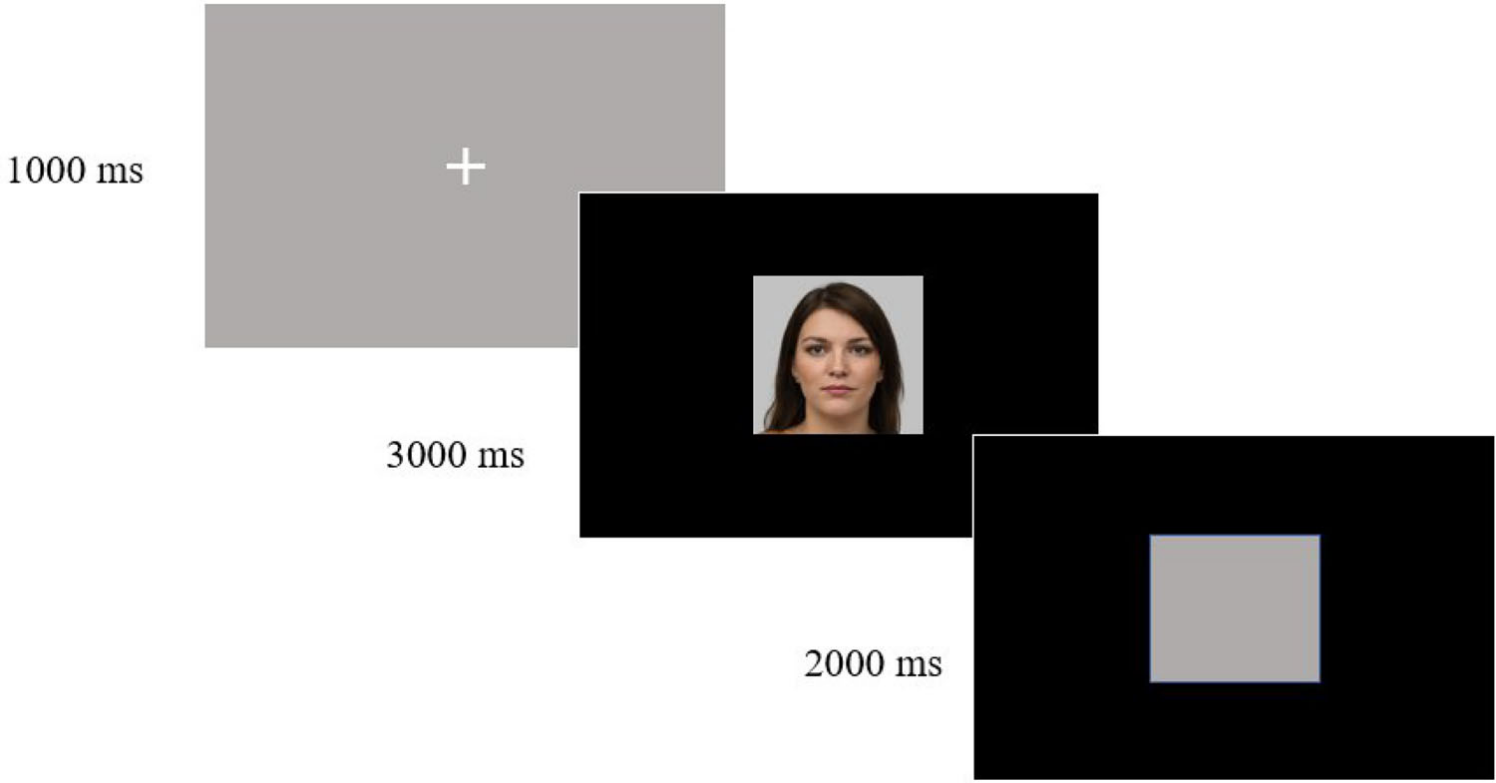
Schematic representation of face‐scanning paradigm. After the presentation of the fixation mark for 1000 ms, the stimuli were shown to the participants for 3000 ms. A gray square was displayed on a black background for 2000 ms between trials.

The fixation mark (white cross) is presented on a gray background for 1000 ms. Stimuli were then presented to the participants for 5000 ms in the practice trials and 3000 ms in the trials. A gray space of the same size as the stimulus was presented on a black background for 2000 ms between trials (Figure 2).

### 2.5 Data analysis

**FIGURE 3:**
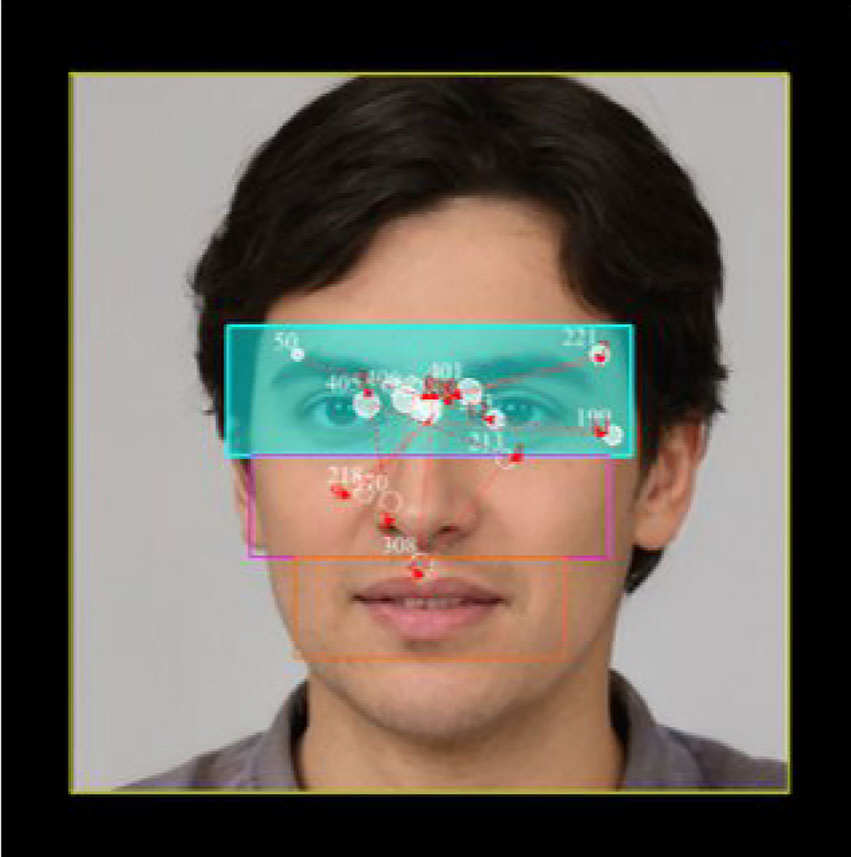
Eye movement during viewing face images was calculated for the entire face and separately for the face, eyes, mouth, and nose areas. The blue area represents the eye area of interest, the pink frame indicates the nose area of interest, and the orange frame corresponds to the mouth area of interest.

In the face‐scanning task, the analyses were carried out by examining the IA of the face separately. Interest areas were determined as eye, nose, and mouth (Figure 3). The number of fixations, mean fixation durations, and the first fixation duration were calculated in the eye, nose, and mouth IA. Analysis of eye movement raw data was performed in the Data Viewer software. Outliers, including blinks and artifacts, were excluded from the analysis.

### 2.6 Statistical analysis

The normality test of eye movement and clinical and demographical parameters were performed by Kolmogorov Smirnov. For variables that were normally distributed, the one‐way ANOVA was employed, whereas the Kruskal–Wallis test was utilized for variables that did not follow a normal distribution. Post hoc tests were carried out using the Bonferroni in one‐way ANOVA.

The repeated measures ANOVA [3 (group: AD, aMCI, and HC) × 3 (IA: eye, nose, and mouth)] was carried out to analyze the eye movements in the eye, nose, and mouth IAs by controlling the between‐subjects group effect. The within‐subject and between‐subjects were determined IAs, and groups, respectively. Post hoc results of statistically significant values for within‐subjects, between‐subjects, and interaction effects were presented to avoid type I errors with Bonferroni. The level of statistical significance was established at *p* < .05.

## 3 RESULTS

### 3.1 Demographical and clinical features

There were no statistical differences in education, age, and sex in all groups. No significant difference was found in the patient group in disease durations.

Significant differences in the neuropsychological test score in AD, aMCI, and HC. The mean and standard deviation of the demographical data and neuropsychological tests, and pairwise comparisons of post hoc tests with the Bonferroni correction of participants are presented in Table 1.

**TABLE 1:** Demographical and clinical features of participants.

	AD (*n* = 31)	aMCI (*n* = 37)	HC (*n* = 33)	*p*	*Statistics*	*Pairwise comparisons*
Education	9.06 ± 5.17	9.22 ± 5.22	11.24 ± 3.0	.103	*F* = 2.453	–
Age	72.81 ± 7.61	70.65 ± 6.47	68.85 ± 6.34	.068	*F* = 3.029	–
Sex (F/M)	15/17	22/15	22/11	.263	*χ* ^2^ = 2.672	–
Disease duration (months)	29.97 ± 21.65	28.59 ± 22.01	–	.820	*t* = −.261	–
GDS	5.59 ± 3.60	8.72 ± 5.14	7.52 ± 4.91	**.036**	***F* = 3.215**	**^a^**
MMSE	22.03 ± 2.81	26.62 ± 1.32	29.09 ± 1.01	**<.001**	***F* = 120.726**	**^a,b,c^**
Digit Span forward	4.0 ± 0.89	4.46 ± 0.65	5.09 ± 0.84	**<.001**	***F* = 15.251**	**^b,c^**
Digit Span backward	2.68 ± 0.90	3.30 ± 0.66	3.97 ± 0.91	**<.001**	***F* = 19.422**	**^a,b,c^**
Trail Making‐B	305.36 ± 90.84	256.20 ± 113.51	135.09 ± 60.75	**<.001**	***F* = 23.192**	**^b,c^**
OVMPT verbal learning	45.0 ± 12.69	76.43 ± 13.07	122.06 ± 10.47	**<.001**	***F* = 326.025**	**^a,b,c^**
OVMPT recall	1.39 ± 2.12	6.49 ± 3.07	13.18 ± 1.55	**<.001**	***F* = 199.578**	**^a,b,c^**
OVMPT recognition	9.74 ± 2.78	7.84 ± 2.70	1.82 ± 1.55	**<.001**	***F* = 95.552**	**^a,b,c^**
RCFT copying	23.68 ± 9.92	31.51 ± 4.67	33.91 ± 3.89	**<.001**	***F* = 21.183**	**^a,b^**
RCFT immediate	4.42 ± 4.15	13.14 ± 6.27	20.17 ± 5.62	**<.001**	***F* = 66.085**	**^a,b,c^**
RCFT delayed	2.48 ± 3.44	12.43 ± 7.12	19.68 ± 6.33	**<.001**	***F* = 67.181**	**^a,b,c^**
RCFT recognition	15.13 ± 2.87	17.95 ± 2.84	18.94 ± 1.91	**<.001**	***F* = 18.173**	**^a,b^**
WCST total errors	56.18 ± 30.03	44.69 ± 24.47	30.10 ± 18.65	**.001**	***F* = 7.106**	**^b^**
WSCT completed category	1.71 ± 2.05	3.13 ± 2.37	5.19 ± 1.32	**<.001**	***F* = 19.113**	**^b,c^**
WCST perseverative response	37.12 ± 32.73	27.97 ± 17.72	19.06 ± 17.29	**.023**	***F* = 3.973**	**^b^**
WCST perseverative errors	29.77 ± 25.70	24.88 ± 14.61	16.42 ± 13.30	**.027**	***F* = 3.804**	**^b^**
Semantic fluency	11.58 ± 4.29	16.14 ± 3.68	23.64 ± 4.24	**<.001**	***F* = 72.249**	**^a,b,c^**
Phonemic fluency	16.83 ± 9.17	25.92 ± 11.70	40.64 ± 17.03	**<.001**	***F* = 26.628**	**^a,b,c^**
Stroop Test	101.26 ± 61.93	59.46 ± 38.18	32.88 ± 7.41	**<.001**	***F* = 21.837**	**^a,b,c^**
Clock Drawing	4.35 ± 3.76	8.11 ± 2.62	9.58 ± 1.48	**<.001**	***F* = 30.487**	**^a,b,c^**
BFRT	33.17 ± 6.20	38.57 ± 5.54	43.42 ± 4.62	**<.001**	***F* = 26.184**	**^a,b,c^**
BJLO	13.5 ± 5.54	15.72 ± 5.0	18.76 ± 4.34	**.003**	***F* = 6.341**	**^b,c^**
BNT	11.48 ± 2.70	13.43 ± 1.18	14.67 ± 0.81	**<.001**	***F* = 26.796**	**^a,b,c^**

*Note*: *F* value refers to one way ANOVA, the *χ* ^2^ value indicates the Chi‐square test, and the *t* value demonstrates the independent samples *t*‐test.

Abbreviations: AD, Alzheimer's disease; aMCI, amnestic mild cognitive impairment; BFRT, Benton Face Recognition Test; BJLO, Benton Judgment of Line Orientation Test; BNT, Boston Naming Test; F, female; GDS, geriatric depression scale; HC, healthy controls; M, male; MMSE, the Mini–mental state examination; OVMPT, Öktem Verbal Memory Processes Test; RCFT, Rey Complex Figure Test; WCST, Wisconsin Card Sorting Test. Statistically significant values are presented in bold.

^a^ Demonstrates the statistical difference between AD and aMCI.

^b^ Demonstrates the statistical difference between AD and HC.

^c^ Demonstrates the statistical difference between aMCI and HC.

### 3.2 Neutral face scanning

#### 3.2.1 Eye, nose, and mouth IAs

There was an IA × GROUP interaction effect on the number of fixations (*F* (2,98) = 3.439, *p* = .020, *η*_p_^2^ = .06). Post hoc results using Bonferroni correction showed that AD patients had fewer eye IA fixations compared to aMCI (*p* = .019) and HC (*p* = .045). Eye movements in the nose and mouth IAs were not different in the groups (*p* > .05).

There was a GROUP main effect on the number of fixations (*F* (2,98) = 4.899, *p* = .009, *η*_p_^2^ = .09). A repeated measure of ANOVA with Bonferroni correction determined that regardless of IA, the number of fixations differed significantly between groups. According to this, AD patients had fewer fixations than HC (*p* = .007). AD and MCI patients and MCI and HC did not differ in the number of fixations (*p* > .05) (Table 2). In addition, there was an IA main effect on the number of fixations (*F* (2,98) = 38.198, *p* < .001, *η*_p_^2^ = .28). Post hoc analysis demonstrated that all participants had more fixations to the eye (4.164 ± 0.259, *p* < .001) and nose IA (3.389 ± 0.160, *p* < .001) than the mouth IA (1.536 ± 0.130). However, there was no significant difference in the number of fixations between the eye and nose IAs (*p* > .05).

**TABLE 2:** Eye movements of the participants in the areas of interest (IA) in the task.

Face scanning	IA	AD (*n* = 31)	aMCI (*n* = 37)	HC (*n* = 33)	*p*‐values			
					Group	IA	Interaction	Pairwise comparisons
Fixation (*n*)	Eye	3.03 ± 2.26	4.80 ± 2.59	4.64 ± 2.87	**.009**	**<.001**	**.020**	AD‐HC *p* = **.045^a^**
	Nose	3.43 ± 1.63	2.91 ± 1.38	3.82 ± 1.78				
	Mouth	1.78 ± 1.65	1.42 ± 1.17	1.39 ± 1.05				AD‐aMCI *p* = **.019^a^**
Fixation durations (ms)	Eye	1050.95 ± 826.41	1560.15 ± 848.47	1358.59 ± 779.45	.087	**<.001**	.082	–
	Nose	1199.29 ± 552.74	1059.60 ± 674.97	1314.67 ± 758.70				
	Mouth	639.37 ± 506.36	528.49 ± 436.61	482.83 ± 370.63				
First fixation duration (ms)	Eye	343.33 ± 141.55	312.98 ± 102.51	303.53 ± 134.29	.735	**<.001**	.432	–
	Nose	341.30 ± 102.59	376.63 ± 158.05	338.95 ± 119.74				
	Mouth	436.24 ± 253.85	401.94 ± 164.39	395.97 ± 276.07				

*Note*: Repeated measures ANOVA results are shown. The symbol “a” indicates pairwise comparisons whose interaction effect is significant in eye IA.

Abbreviations: AD, Alzheimer's disease; aMCI, amnestic mild cognitive impairment; HC, healthy controls. Statistically significant values are presented in bold.

There was no main GROUP effect (*F* (2,98) = 2.499, *p* = .087) and IA × GROUP interaction effect on mean fixation durations (*F* (2,98) = 2.245, *p* = .082). However, there was a main IA effect on fixation durations (*F* (2,98) = 28.261, *p* < .001, *η*_p_^2^ = .22). The fixation durations to the eye (1323.236 ± 81.782 ms, *p* < .001) and nose (1191.190 ± 66.843 ms, *p* < .001) IAs were significantly longer than that to the mouth (550.234 ± 43.874 ms) IA in all participants. The mean fixation durations to the eye and nose IAs were not statistically different in all participants (*p* > .05).

There were no main GROUP (*F* (2,98) = .308, *p* = .735) and IA × GROUP interaction effects on the first fixation duration (*F* (2,98) = .924, *p* = .432). However, there was a main IA effect on the first fixation duration (*F* (2,98) = 15.403, *p* < .001). The first fixation duration for the eye (319.948 ± 12.649, *p* < .001) and nose (352.216 ± 13.161, *p* = .007) IAs was significantly shorter than that for the mouth IA (411.180 ± 23.517) in all participants. Moreover, the first fixation duration to the eye IA was significantly shorter than the nose IA in all participants (*p* = .013). Table 2 shows the mean and standard deviation values of eye movement events in IAs in the groups.

**FIGURE 4:**
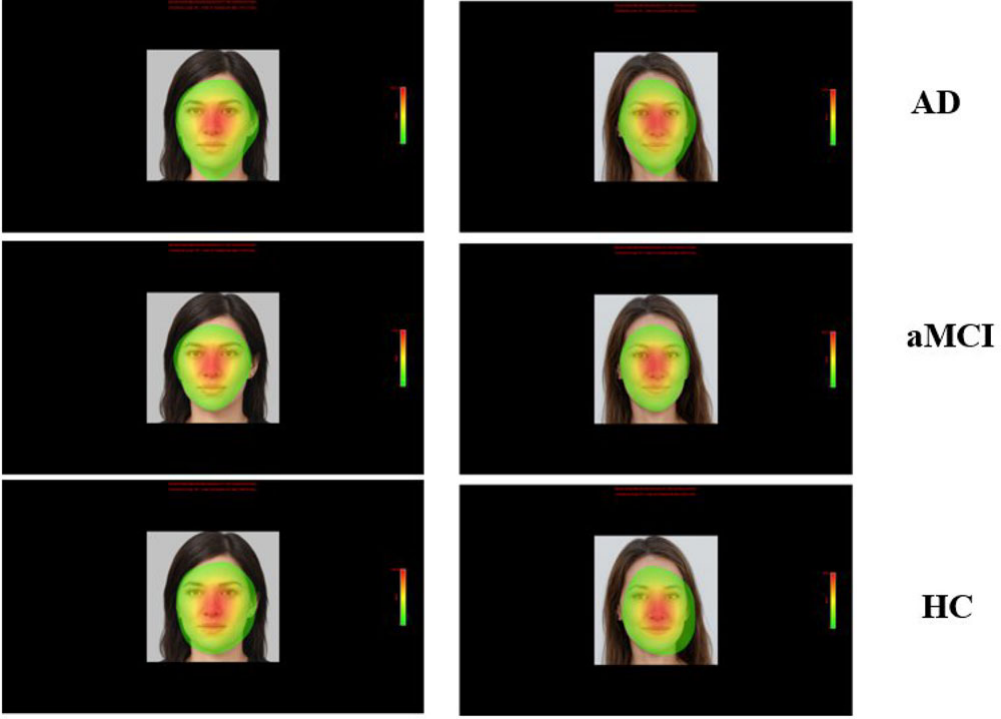
The heat map display of fixation frequency on stimuli. Red represents the most frequently fixated areas, and the green represents the least fixation areas in the heap map.

The heat map representing the fixation frequency of the participants is shown in Figure 4. This figure was created by combining the fixation of AD, aMCI, and HC on facial stimuli in the EyeLink 1000 Plus Data Viewer software (SR Research).

**FIGURE 5:**
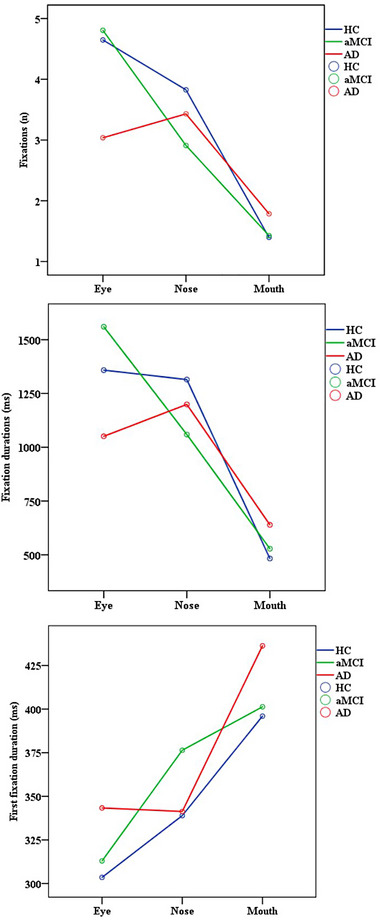
Eye movement measurements of Alzheimer's disease (AD), amnestic mild cognitive impairment (aMCI), and healthy controls (HC) in the eye, mouth, and nose areas of interest.

The line graph of the number of fixations, fixation durations, and first fixation duration in IAs and groups was presented in Figure 5.

## 4 DISCUSSION

The fundamental finding of this study was that AD looked at the eye IA on faces less than aMCI and healthy older individuals. The secondary finding of our study was that all participants regardless of patient and control groups looked at the eye and nose IAs more and longer than the mouth IA.

When recognizing faces, describing face expressions, or distinguishing gender, people use consistent visual information from the eye region through both explicit and implicit attentional processes (Emery, 2000; Loomis et al., 2008). Studies with healthy adults have reported typical eye movement patterns focusing primarily on the eyes, nose, and mouth regions, in addition to individual differences (Buswell, 1935; Fraser et al., 1990; Haig, 1985; 1986; Tanaka & Farah, 1993; Yarbus, 1967). Our study also confirmed these findings. Regardless of the group, more fixations were found in the eye and nose IA than in the mouth IA. Significant evidence of eye dominance in face identification indicates that adult humans can identify and distinguish faces only from their eyes. It is stated that it can provide information in the first construction of categorical information about the eye and its surrounding regions, faces, and the gaze may have special importance in social communication (Hoffman & Haxby, 2000; Lim et al., 2011; O'Donnell & Bruce, 2001). It was stated that the behavior of people to look into the eyes of others is a social behavior learned from an early age (Farroni et al., 2002).

Although the eye is the most important part of the face, in our study, a decrease was found in the number of fixations and the duration of fixations in the eye IA of AD patients. Consistent with our hypothesis, AD patients scanned face images superficially and focused less on informative parts such as the eye and nose. The inability to pay attention to face IAs may cause face perception difficulties in individuals with AD. The decrease in the gaze toward the face in AD patients may be related to face‐processing difficulties. Impairments in facial expression recognition and facial memory have been shown in many studies of AD (Güntekin et al., 2019; Lavallée et al., 2016; Mazzi et al., 2020; Seelye et al., 2009; Spoletini et al., 2008; Werheid & Clare, 2007). These impairments may be related to the inefficient face‐scanning found in our study. In this study, no significantly different eye movement models were found in viewing face images between aMCI patients and HC. A study investigated eye movement patterns in both face tasks of aMCI patients. In this study, it was found that patients with aMCI gaze at the mouth region more than controls during face processing (Kawagoe et al., 2017). The results of this study are different from our study. This may be related to the different stimuli used in the experiment. Our stimuli also included hair, neck, and background, while Kawagoe et al. (2017) used a face image containing only the face circle. This may have caused the fixations to spread over a wider area in our study. Although our findings are not consistent, the study of Kawagoe et al. (2017) is the only study we know of to record eye movements while viewing face images in aMCI. The findings of this study form the basis for the eye movement literature in face processing in aMCI. Moreover, there is no study examining eye movements during face processing in AD so far. Our current study is the only study in which the facial perception of both AD and aMCI patients was examined by eye movements.

The brain scans of aMCI participants taken 3 years before AD diagnosis displayed a significant decrease in gray matter in the medial temporal lobes, such as the amygdala and the fusiform gyrus (Whitwell et al., 2007). According to these findings, the initial signs of AD appeared in the anterior medial temporal lobe and the fusiform gyrus. Evidence indicates that facial processing is relatively focused on the ocular region. Neurodegeneration could cause specific susceptibility to the face‐processing network, impairing facial perception (Lim et al., 2011). Functional neuroimaging studies showed lower activation of fusiform area related to face processing in AD, and MCI compared to controls (Carp et al., 2011; Lee et al., 2011; Park et al., 2012; Rieck et al., 2015; Voss et al., 2008). One study found face processing early changes as a visuospatial ability in aMCI patients (Lim et al., 2011). They also suggested that face‐processing skills should also be evaluated as a behavioral sign for the early phases of dementia.

Up to the present, this is the first study to examine eye movement models during face viewing in AD aMCI. Studies examining eye movement patterns during face perception in AD will contribute to distinguishing face‐specific difficulties.

## AUTHOR CONTRIBUTIONS

Hatice Eraslan Boz, Koray Koçoğlu and Gülden Akdal designed the study. Gülden Akdal was responsible for the clinical evaluation of the participants and their assignment to the groups. Müge Akkoyun, Işil Yağmur Tüfekçi, and Merve Ekin were responsible for eye movement recordings and raw data. Hatice Eraslan Boz is responsible for performing neuropsychological assessments. Hatice Eraslan Boz, Koray Koçoğlu and Müge Akkoyun analyzed eye data. All authors contributed to the initial draft of the manuscript. Hatice Eraslan Boz, Müge Akkoyun and Gülden Akdal decided on the final version of the manuscript after revisions.

## CONFLICT OF INTEREST STATEMENT

The authors declare no conflicts of interest.

### PEER REVIEW

The peer review history for this article is available at https://publons.com/publon/10.1002/brb3.3232.

## Data Availability

The data that support the findings of this study are available from the corresponding author upon reasonable request.
